# The Antimicrobial Peptide Human Beta-Defensin-3 Is Induced by Platelet-Released Growth Factors in Primary Keratinocytes

**DOI:** 10.1155/2017/6157491

**Published:** 2017-07-25

**Authors:** Andreas Bayer, Justus Lammel, Mersedeh Tohidnezhad, Sebastian Lippross, Peter Behrendt, Tim Klüter, Thomas Pufe, Jochen Cremer, Holger Jahr, Franziska Rademacher, Regine Gläser, Jürgen Harder

**Affiliations:** ^1^Department of Heart and Vascular Surgery, University Hospital of Schleswig-Holstein, Campus Kiel, Arnold-Heller Straße 3, Haus 26, 24105 Kiel, Germany; ^2^Department of Dermatology, University Hospital of Schleswig-Holstein, Campus Kiel, Schittenhelmstr. 7, 24105 Kiel, Germany; ^3^Institute of Anatomy and Cell Biology, RWTH University of Aachen, Wendlingweg 2, 52072 Aachen, Germany; ^4^Department of Traumatology, University Hospital of Schleswig-Holstein, Campus Kiel, Arnold-Heller Straße 3, Haus 18, 24105 Kiel, Germany; ^5^Department of Orthopaedics, Aachen University Hospital, Pauwelsstraße 30, 52074 Aachen, Germany

## Abstract

Platelet-released growth factors (PRGF) and its related clinically used formulations (e.g., Vivostat Platelet-Rich Fibrin (PRF®)) contain a variety of chemokines, cytokines, and growth factors and are therefore used to support healing of chronic, hard-to-heal, or infected wounds. Human beta-defensin-3 (hBD-3) is an antimicrobial peptide inducibly expressed in human keratinocytes especially upon wounding. The potent antimicrobial activity of hBD-3 together with its wound closure-promoting activities suggests that hBD-3 may play a crucial role in wound healing. Therefore, we analyzed the influence of PRGF on hBD-3 expression in human primary keratinocytes *in vitro*. In addition, we investigated the influence of Vivostat PRF on hBD-3 expression in artificially generated human skin wounds *in vivo*. PRGF treatment of primary keratinocytes induced a significant, concentration- and time-dependent increase in hBD-3 gene expression which was partially mediated by the epidermal growth factor receptor (EGFR). In line with these cell culture data, *in vivo* experiments revealed an enhanced hBD-3 expression in experimentally produced human wounds after the treatment with Vivostat PRF. Thus, the induction of hBD-3 may contribute to the beneficial effects of thrombocyte concentrate lysates in the treatment of chronic or infected wounds.

## 1. Introduction

Platelet-released growth factors (PRGF) is a thrombocyte concentrate lysate comprising a multitude of chemokines, cytokines, and growth factors [1–5]. The *in vitro* capacities of PRGF to stimulate cell proliferation and tissue regeneration, to modify cell and tissue differentiation, and to support angiogenesis [6–12] suggest that PRGF may serve as an optimal therapeutic tool for the treatment of chronic or complicated wounds. Accordingly, its clinically related formulation Vivostat PRF has already been used successfully to support healing of patients' hard-to-heal wounds *in vivo* [13]. However, the underlying mechanisms of these observed beneficial *in vivo* effects are not well understood.

In general, one possible reason for a malfunctioning wound healing process of the skin is a local wound infection with potential pathogenic bacterial species. In this situation, human keratinocytes establish a chemical defense system based on the production of antimicrobial peptides, for example, human beta-defensin- (hBD-) 2 and hBD-3 to defeat these microbial threats [14–16]. Recently, we demonstrated that PRGF induces the antimicrobial peptide hBD-2 in primary keratinocytes [17] showing for the first time that PRGF is able to induce the expression of antimicrobial peptides in cultured keratinocytes as well as in wounded skin.

Human beta-defensin-3 (hBD-3) is another important antimicrobial peptide involved in cutaneous defense and was originally isolated from lesional psoriatic skin [18]. hBD-3 is expressed in many epithelia (e.g., skin, respiratory tract, digestive tract, and genitourinary tract) [14, 15, 19–21]. Its expression was shown to be induced by cytokines or microbial stimuli [16, 22]. Hirsch et al. showed that hBD-3 expression significantly promotes wound closure in *S. aureus*-infected diabetic wounds in a preclinical large-animal model [23]. Furthermore, a tenfold reduction in bacterial growth on day 4 was detected in this study indicating that human beta-defensin-3 may play a major role at least in diabetic wound healing and wound infections [23]. As these data suggest an important role of hBD-3 for the wound healing process, we analyzed the influence of PRGF on hBD-3 expression in human primary keratinocytes *in vitro* and of Vivostat PRF on hBD-3 expression in artificially generated human skin wounds *in vivo*.

## 2. Materials and Methods

### 2.1. Preparation of PRGF

PRGF was isolated from platelet lysates of several freshly donated human thrombocyte concentrates received from the Institute of Transfusion Medicine, University of Schleswig-Holstein, Campus Kiel. These thrombocyte concentrates were centrifuged for 10 minutes at 2000*g*. The thrombocyte pellet was washed twice with sodium citrate buffer (0.11 mM, pH 5.5, 37°C) and centrifuged again for 10 min at 2000*g*. Afterwards, the thrombocytes were resuspended in half the volume of the initial thrombocyte concentrate volume using Keratinocyte Growth Medium 2 (KGM-2, PromoCell, Heidelberg, Germany) without supplements. The thrombocytes were lysed on ice by ultrasound and stored at −80°C for 24 hours and again lysed by ultrasound and stored at −80°C for 24 hours. Subsequently, cell debris was removed by centrifugation for 1 minute at 18,000*g*, and the remaining supernatant (now named PRGF) was stored in aliquots at −20°C.

### 2.2. Culture and Stimulation of Primary Human Keratinocytes

Foreskin-derived primary human keratinocytes pooled from different individuals (PromoCell, Heidelberg, Germany) were cultured in KGM-2 at 37°C in a humidified atmosphere with 5% CO_2_. Stimulation of the keratinocytes with the indicated concentration of PRGF was done in 12-well tissue culture plates (BD Biosciences, Franklin Lakes, New Jersey) when the cells reached a confluence of 90–100%. We used the EGFR-blocking antibody cetuximab (Merck, Darmstadt, Germany) at a concentration of 20 *μ*g/ml and the IL-6 receptor-blocking antibody tocilizumab (Hoffmann-La Roche, Basel, Switzerland) at a concentration of 50 *μ*g/ml to analyze the influence of the EGFR and the IL-6 receptor on hBD-3 induction. After stimulation, we harvested the supernatants for ELISA and washed the cells with 1 ml per well of PBS before RNA isolation.

### 2.3. RNA Isolation and cDNA Synthesis

To isolate total RNA, keratinocytes derived from one well of a 12-well plate were lysed with 500 *μ*l Crystal RNAmagic reagent, and total RNA was isolated as recommended in the supplier's protocol (BiolabProducts, Bebensee, Germany). Determination of RNA quantity and quality was carried out photometrically using a NanoDrop device (Peqlab, Erlangen, Germany). 1 *μ*g total RNA served as a template in a reverse transcription reaction using oligo-dT-primers and 50 units Maxima Reverse Transcriptase (Thermo Fisher Scientific, Waltham, USA) according to the manufacturer's protocol. The resulting cDNA was stored at −20°C until use.

### 2.4. Real-Time PCR

For real-time PCR analyses, we used a fluorescence temperature cycler (StepOnePlus, Life Technologies) as previously described [24]. The following intron-spanning primers were used: hBD-3—5′-TGT TCC TGT-3′ (forward primer) and 5′-CGC CTC TGA CTC TGC AAT AA-3′ (reverse primer), and RPL38 (ribosomal protein L38)—5′-TCA AGG ACT TCC TGC TCA CA-3′ (forward primer) and 5′-AAA GGT ATC TGC TGC ATC GAA-3′ (reverse primer). Serial dilutions of cDNA served as standards to obtain standard curves for relative quantification. Relative gene expression is depicted as the ratio between hBD-3 expression and expression of the house keeping gene RPL38.

### 2.5. Luciferase Reporter Assays

To analyze activation of the hBD-3 promoter activity, a firefly luciferase plasmid containing 2484 bp of the human hBD-3 promoter was used. The hBD-3 promoter region was amplified using the primers 5′-AGC CTC GAG TGC AGT TCC AAG TGC TGT GAC-3′ and 5′-CAG AAG CTT GGA TGA AAA GGT GTG CTT GGT C-3′. Both primers contain recognition sites for the restriction enzymes XhoI and HindIII, respectively, to facilitate subsequent cloning into the promoterless pGL3-basic firefly luciferase vector (Promega, Madison, WI). Verification of the correct insertion of the hBD-3 promoter region into the pGL3-basic plasmid was done by sequencing. To analyze hBD-3 promoter activity, 0.5 *μ*g of the hBD-3 firefly promoter plasmid was transfected together with 0.05 *μ*g of an internal control *Renilla* luciferase expression plasmid (pGL4.74[hRluc/TK], Promega) in primary keratinocytes (70–90% confluence) using the transfection reagent FuGENE HD (Promega). After 6 hours, the transfection medium was removed and replaced by fresh medium. After additional 20–24 hours, cells were stimulated with PRGF (1 : 10 diluted in KGM-2) for 24 hours. Luciferase activity was analyzed using the Dual-Luciferase Assay System (Promega) on a TD-20/20 luminometer (Turner Design, Sunnyvale, CA). Relative promoter activity was calculated as the ratio between firefly and *Renilla* luciferase activity.

### 2.6. Analyses of the Influence of Vivostat PRF on the hBD-3 Expression in Keratinocytes In Vivo

We generated bilateral gluteal wounds in five male healthy volunteers by a punch biopsy (Ø 4 mm) after local anesthesia. Immediately, we treated the wounds with either NaCl 0.9% (left) or freshly produced Vivostat PRF (right). The remaining Vivostat PRF was stored at −20°C. After 5 days, we thawed the frozen Vivostat PRF and repeated the wound treatment. On day 10, we set local anesthesia, resected the bilateral wound areas by punch biopsies (Ø 6 mm), and bisected the received skin biopsies. One-half of the biopsy was used for RNA isolation using the RNAeasy kit (Qiagen, Hilden, Germany). Reverse transcription of the RNA and real-time PCR were performed as described above. The second half of the biopsy was embedded in paraffin and used for hBD-3 immunohistology as described recently [21]. This study was approved by the university committee for ethical affairs, Kiel (AZ A 115/13), in accordance with the Helsinki guidelines. All participants included in this investigation provided written informed consent.

### 2.7. Statistics

GraphPad Prism 6.07 was used for statistical analysis and was carried out by Student's *t*-test. A *p* value of <0.05 was considered statistically significant.

## 3. Results

### 3.1. Stimulation of Human Primary Keratinocytes with PRGF Induces hBD-3 Expression

**Figure 1:**
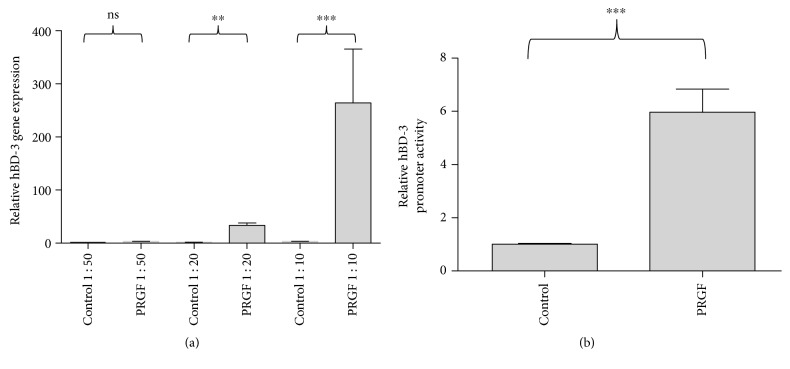
Expression of hBD-3 is induced in primary human keratinocytes by PRGF treatment. (a) Primary keratinocytes were treated with different PRGF concentrations (PRGF 1 : 50, 1 : 20, and 1 : 10 diluted in cell culture medium) for 24 hours. Subsequently, RNA was isolated and reverse-transcribed in cDNA, and hBD-3 gene expression was analyzed by real-time PCR. (b) HBD-3 promoter activation was analyzed in keratinocytes transfected with a hBD-3 promoter luciferase plasmid after stimulation with PRGF (diluted to 1 : 10 in cell culture medium) for 24 hours (^∗∗^*p* < 0.01, ^∗∗∗^*p* < 0.001, ns = not significant, Student's *t*-test).

We stimulated human primary keratinocytes with different concentrations of PRGF for 24 hours to analyze PRGF-mediated induction of hBD-3 gene expression in human primary keratinocytes. PRGF stimulation caused a significant induction of hBD-3 gene expression (Figure 1(a)). In concordance with hBD-3 gene expression, PRGF induced also activation of the hBD-3 promoter as analyzed by an hBD-3 reporter luciferase plasmid (Figure 1(b)).

### 3.2. The PRGF-Mediated hBD-3 Induction in Primary Human Keratinocytes Is Time-Dependent

**Figure 2:**
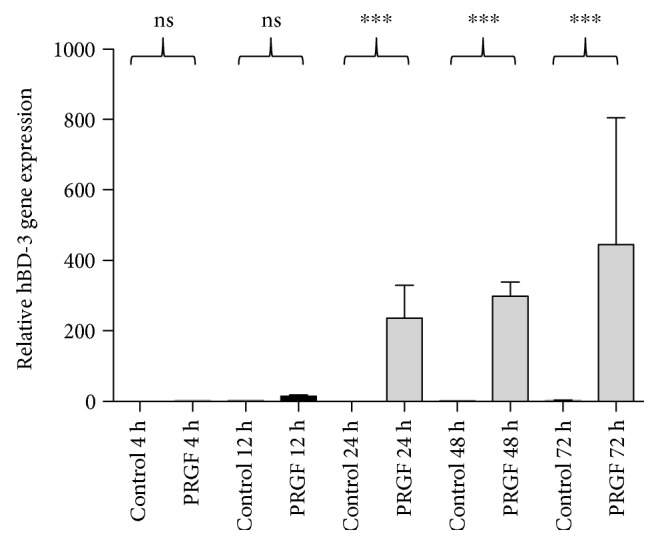
hBD-3 mRNA induction by PRGF is time-dependent. Primary keratinocytes were stimulated with PRGF (1 : 10 diluted in cell culture medium) for different time periods (4, 12, 24, 48, and 72 hours), and hBD-3 gene expression was analyzed by real-time PCR (^∗∗∗^*p* < 0.001, ns = not significant, Student's *t*-test).

We analyzed hBD-3 gene expression after 4, 12, 24, 48, and 72 hours of PRGF stimulation to assess the time kinetic of PRGF-mediated hBD-3 induction. hBD-3 was significantly induced in primary keratinocytes after 24 hours of PRGF stimulation (Figure 2). The high hBD-3 induction persisted after 48–72 hours of treatment periods (Figure 2).

### 3.3. The PRGF-Induced hBD-3 Expression in Primary Keratinocytes Is Partially Mediated by the Epidermal Growth Factor Receptor (EGFR)

**Figure 3:**
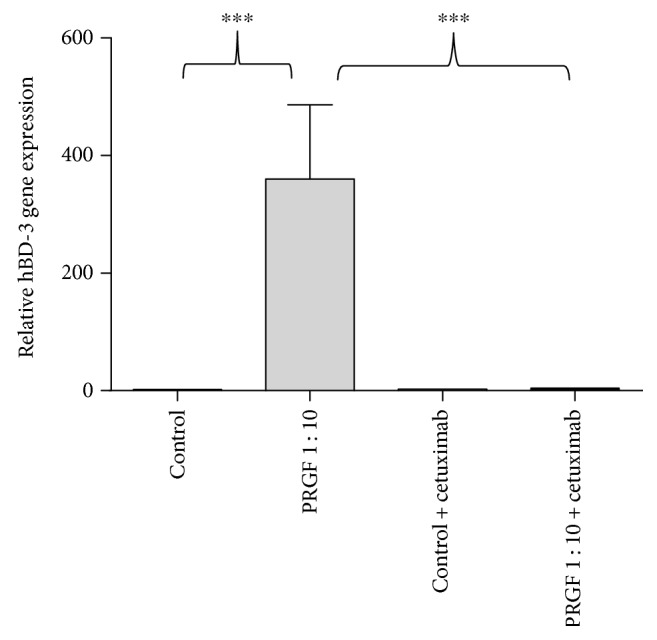
The epidermal growth factor receptor (EGFR) partially mediated the hBD-3 gene induction in keratinocytes stimulated with PRGF. To analyze the influence of the EGFR on the PRGF-mediated hBD-3 induction, human primary keratinocytes were treated with PRGF (1 : 10 diluted in cell culture medium) in the presence or absence of the EGFR-blocking antibody cetuximab. HBD-3 gene expression was analyzed by real-time PCR (^∗∗∗^*p* < 0.001, ns = not significant, Student's *t*-test).

We used a specific monoclonal EGFR-blocking antibody (cetuximab) to investigate the possible influence of the EGF receptor on the PRGF-mediated induction of hBD-3 in primary human keratinocytes. Treatment of PRGF-stimulated keratinocytes with cetuximab caused a significant decrease in the PRGF-induced hBD-3 gene expression (Figure 3).

### 3.4. The Influence of the Interleukin-6 Receptor (IL-6R) on the PRGF-Induced hBD-3 Expression in Human Primary Keratinocytes

**Figure 4:**
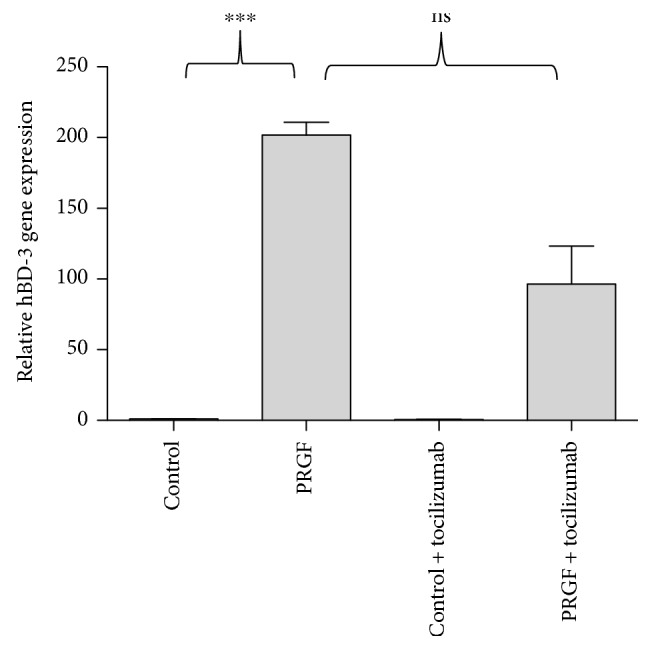
IL-6 signaling seems to play only a marginal role for the observed PRGF-induced hBD-3 expression. We stimulated primary human keratinocytes with PRGF (1 : 10 diluted in cell culture medium) and used the IL-6 receptor-blocking antibody tocilizumab (50 *μ*g/ml) to analyze the influence of IL-6 signaling on PRGF-mediated hBD-3 gene induction. HBD-3 gene expression was analyzed by real-time PCR (^∗∗∗^*p* < 0.001, ns = not significant, Student's *t*-test).

Since we have previously shown that PRGF rapidly induced IL-6 gene expression in primary human keratinocytes [17], we investigated the possible role of IL-6 on the PRGF-mediated hBD-3 induction in human keratinocytes by blocking the IL-6 receptor with the IL-6 receptor-neutralizing antibody tocilizumab. Treatment of keratinocytes with tocilizumab partially blocked the PRGF-induced hBD-3 gene expression; however, this was statistically not significant (Figure 4).

### 3.5. Vivostat PRF Treatment of Cutaneous Wounds Resulted in an Increased Expression of hBD-3 in Human Epidermis In Vivo

**Figure 5:**
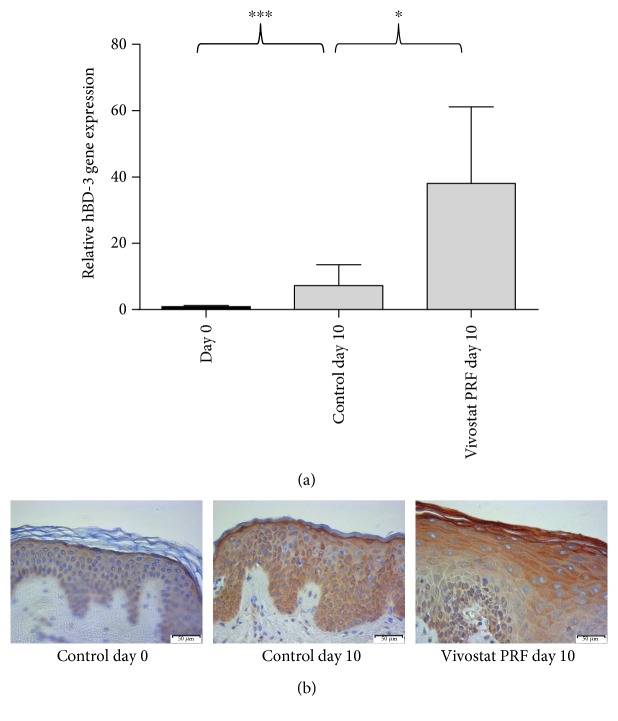
HBD-3 is induced by Vivostat PRF treatment *in vivo*. In the *in vivo* study, we observed a strong hBD-3 gene induction by artificial wound generation which was significantly increased by local wound therapy by Vivostat PRF (a) (^∗^*p* < 0.05, ^∗∗∗^*p* < 0.001, Student's *t*-test). (b) Correspondingly, using immunohistochemistry, we observed a strong hBD-3 induction in the uppermost epidermal layers of artificially generated skin wounds (control day 10) that was intensified by a local Vivostat PRF wound treatment on day 0 and day 5 of the conducted study. Scale bars represent 50 *μ*m.

To assess whether the *in vitro* results are transferable into the *in vivo* situation, we sought to determine the influence of Vivostat PRF on the hBD-3 expression in experimentally generated gluteal skin wounds of five male healthy volunteers. These wounds were generated bilaterally by 4 mm punch biopsies. On day zero and day five, left wounds were treated with NaCl 0.9% as a control whereas right wounds were treated with Vivostat PRF. On day ten, bilateral wound areas were resected by 6 mm biopsy punches, bisected, and used either for RNA isolation, cDNA synthesis, and real-time PCR analysis or for immunohistological analyses of hBD-3 expression. This *in vivo* study revealed a significant induction of hBD-3 gene expression in Vivostat PRF-treated wounds (Figure 5(a)) which was accompanied by an increased hBD-3 staining in the Vivostat PRF-treated wounds compared to the control wounds (Figure 5(b)).

## 4. Discussion

In western countries, about 3% of the population suffer from chronic leg ulcers causing an immense personal, financial (approximately 2.5% of total healthcare budgets in Europe and America), and social burden [25]. Therapeutic options for the therapy of chronic, hard-to-heal wounds are rare, expensive, and often insufficient. Frequently, worsening hard-to-heal wounds result in patients' minor or even major extremity amputation.

Thrombocyte concentrate lysates generally contain a variety of chemokines, cytokines, and growth factors [1–5] which are able to stimulate cell proliferation and tissue regeneration, to modify cell and tissue differentiation, and to support angiogenesis. This qualifies thrombocyte concentrate lysates as a potential therapeutic tool for the treatment of hard-to-heal wounds *in vivo*. Indeed, the regenerative, reparative, and angiogenetic potential of thrombocyte concentrate lysates leads to an increased use in many medical disciplines [26–30]. Surprisingly, only a few studies about treatment of patients' chronic skin ulcers with thrombocyte concentrate lysates (e.g., Vivostat PRF) are published. In a small study of chronic skin ulcers, superior healing rates by local treatment with a platelet concentrate lysate compared with a standard control treatment were revealed [31]. Local PRGF treatment was also reported to heal a chronic severe mal performant foot ulcer in a diabetic patient [32]. Steenvoorde et al. described the use of Vivostat PRF on a variety of hard-to-heal wounds which caused complete wound healing or a significant reduction in wound diameter in a majority of treated patients [13]. Using Vivostat PRF for the local treatment of chronic or complicated wounds, we observed in our own department a complete wound healing in 32% and a reduction in the wound diameter in 26% of these patients (*n* = 50).

Despite these positive clinical experiences with thrombocyte concentrate lysates for the therapy of chronic skin ulcers, only little is known about possible involved mechanisms [8, 11, 12, 33–35]. As we have recently shown that platelet-released growth factors induce the antimicrobial peptide human beta-defensin-2 in primary keratinocytes [17], we now aimed to analyze the influence of PRGF on the expression of hBD-3 in keratinocytes, because hBD-3 belongs to the innate epithelial defense system and seems to play an important role in the wound healing process [22, 23]. Human beta-defensin-3 (hBD-3) was originally isolated from lesional psoriatic skin extracts [18] and is inducibly expressed in many epithelia as the skin and respiratory, digestive, and genitourinary tract [16, 19, 21, 36, 37]. In contrast to hBD-2, hBD-3 is a multifunctional peptide. Compared to hBD-2, hBD-3 offers broader antimicrobial effects [15, 19, 20, 38, 39] even against multiresistant bacterial strains [40], fungi, and viruses [41, 42]. Its functional relevance was demonstrated by Kisich et al. who showed that the constitutive capacity of human keratinocytes to kill *Staphylococcus aureus* is dependent on hBD-3 [38]. Besides its antimicrobial and wound closure properties, hBD-3 activates different immune and inflammatory cells and stimulates epidermal keratinocyte migration and proliferation and production of proinflammatory cytokines and chemokines [43]. Therefore, hBD-3 displays a key mediator in skin immunity and wound healing.

In our study, we noticed a significant concentration-dependent induction of hBD-3 gene expression in PRGF-treated keratinocytes. Moreover, hBD-3 gene induction in keratinocytes by PRGF treatment was time-dependent with the highest levels after 24–72 hours.

These *in vitro* data were consistent with our *in vivo* experiments where we depicted that treatment of cutaneous wounds with Vivostat PRF caused a strong induction of hBD-3 in the keratinocytes *in vivo*. In summary, the results of our experiments identify PRGF *in vitro* and Vivostat PRF *in vivo* as a potent activator of epidermal hBD-3 expression.

It is known that hBD-3 can be induced via activation of the epidermal growth factor receptor (EGFR) [39, 44]. Therefore, we hypothesized that PRGF induces hBD-3 via activation of the EGFR pathway similarly as recently reported for hBD-2 [17]. To prove this hypothesis, we used an antibody (cetuximab) directed against the EGFR. Blocking the EGFR significantly inhibited the induction of hBD-3 in keratinocytes by PRGF indicating an essential role of the EGFR in the PRGF-mediated hBD-3 induction. This is in concordance with a study demonstrating that stimulation of keratinocytes with EGFR ligands strongly induced hBD-3 expression [45]. It remains to be shown whether the observed PRGF-induced hBD-3 induction may be directly mediated by EGFR ligands present in the PRGF.

Another study reported that expression of hBD-3 in keratinocytes is induced upon wounding in an EGFR-dependent manner [46]. The critical role of hBD-3 in wound healing is further documented by studies reporting on the capacity of hBD-3 to promote wound healing [23] and to control the growth of wound-related pathogens including multiresistant strains [38, 40]. Since bacterial biofilms exhibit a negative effect on wound healing, it is of special interest that hBD-3 is able to reduce bacterial biofilm development [47, 48]. These observations suggest that hBD-3 induction by PRGF may contribute to the observed positive clinical effect of thrombocyte concentrate lysates on human wound healing. Its capability to induce antimicrobial peptides such as hBD-3 and hBD-2 [17] may also endow thrombocyte concentrate lysate with the potential to protect from wound infections.

Interleukin-6 (IL-6) is a multifunctional cytokine produced by various cell types that also influences various cell types and has multiple biological activities [49]. It regulates immune responses, hematopoiesis, acute -phase responses, and inflammation and plays a critical role in the pathogenesis of various immune processes and epithelial immune responses [50–54]. Since we recently demonstrated that PRGF induced the expression of IL-6 in human primary keratinocytes [17], we sought to evaluate if the observed PRGF-mediated hBD-3 induction is IL-6-dependent. Therefore, we used an antibody (tocilizumab) directed against the IL-6 receptor to inhibit the IL-6 signaling pathway. Blocking the IL-6 receptor resulted in a reduced hBD-3 gene induction, but this reduction achieved no statistical significance. Thus, in contrast to hBD-2 [17], IL-6 signaling seems to play only a marginal—if any—role for the observed PRGF-induced hBD-3 expression. This is in concordance with that of studies demonstrating that IL-6 has no major influence on hBD-3 expression in keratinocytes [55] and underlines the biological differences between hBD-2 and hBD-3.

## 5. Conclusion

We identified PRGF *in vitro* as a potent inducer of hBD-3 in primary keratinocytes. The induced hBD-3 expression was concentration- and time-dependent and partially mediated by the EGFR. In line with these *in vitro* data, our human *in vivo* study revealed an upregulation of hBD-3 expression in experimentally generated wounds upon treatment with Vivostat PRF. The PRGF- and Vivostat PRF-induced hBD-3 expression in primary human keratinocytes could be one of the mechanisms leading to the improved clinical outcome often seen after treatment of chronic and/or infected wounds with Vivostat PRF.
